# Patterned feeding experience for preterm infants: study protocol for a randomized controlled trial

**DOI:** 10.1186/s13063-015-0781-3

**Published:** 2015-06-04

**Authors:** Rita H. Pickler, Paul A. Wetzel, Jareen Meinzen-Derr, Heather L. Tubbs-Cooley, Margo Moore

**Affiliations:** Cincinnati Children’s Hospital Medical Center, 3333 Burnet Avenue, Cincinnati, OH 45229 USA; Virginia Commonwealth University, 401 West Main Street, PO Box 843067, Richmond, VA USA

**Keywords:** Preterm infant, Infant feeding, Neonatal care, Nursing care, Neurodevelopment, Cognitive development, Clinical outcomes, Length of stay, Feeding experience

## Abstract

**Background:**

Neurobehavioral disabilities occur in 5–15 % of preterm infants with an estimated 50–70 % of very low birth weight preterm infants experiencing later dysfunction, including cognitive, behavioral, and social delays that often persist into adulthood. Factors implicated in poor neurobehavioral and developmental outcomes are hospitalization in the neonatal intensive care unit (NICU) and inconsistent caregiving patterns. Although much underlying brain damage occurs in utero or shortly after birth, neuroprotective strategies can stop lesions from progressing, particularly when these strategies are used during the most sensitive periods of neural plasticity occurring months before term age. The purpose of this randomized trial is to test the effect of a patterned feeding experience on preterm infants’ neurobehavioral organization and development, cognitive function, and clinical outcomes.

**Methods:**

This trial uses an experimental, longitudinal, 2-group design with 120 preterm infants. Infants are enrolled within the first week of life and randomized to an experimental group receiving a patterned feeding experience from the first gavage feeding through discharge or to a control group receiving usual feeding care experience. The intervention involves a continuity of tactile experiences associated with feeding to train and build neuronal networks supportive of normal infant feeding experience. Primary outcomes are neurobehavioral organization as measured by Neurobehavioral Assessment of the Preterm Infant at 3 time points: the transition to oral feedings, NICU discharge, and 2 months corrected age. Secondary aims are cognitive function measured using the Bayley Scales of Infant and Toddler Development, Third Edition at 6 months corrected age, neurobehavioral development (sucking organization, feeding performance, and heart rate variability), and clinical outcomes (length of NICU stay and time to full oral feeding). The potential effects of demographic and biobehavioral factors (perinatal events and conditions of maternal or fetal/newborn origin and immunologic and genetic biomarkers) on the outcome variables will also be considered.

**Discussion:**

Theoretically, the intervention provided at a critical time in neurologic system development and associated with a recurring event (feeding) should enhance neural connections that may be important for later development, particularly language and other cognitive and neurobehavioral organization skills.

**Trial registration:**

NCT01577615 11 April 2012.

**Electronic supplementary material:**

The online version of this article (doi:10.1186/s13063-015-0781-3) contains supplementary material, which is available to authorized users.

## Background

Despite a survival rate that exceeds 85 %, between 5–15 % of very preterm infants born at less than 28 weeks post-menstrual age (PMA) will have long-term neurodevelopmental consequences of prematurity including cerebral palsy and severe neurosensory impairment [1]. Moreover, an estimated 50–70 % of very low birth weight preterm infants (≤1500 g) have late onset neurodevelopmental dysfunction, including cognitive, behavioral, and social delays [2]. These dysfunctions often persist into adulthood [3], making preterm birth one of the most costly and devastating of all health events [4].

A major reason for increased risk of poor neurodevelopmental outcomes is the structural differentiation of the central nervous system (CNS) that occurs rapidly between 23 and 32 weeks of gestation [5]. In a term pregnancy, this differentiation occurs in utero, with a “system” that is designed by nature to support the biologic processes that are occurring. However, the neurologic system of infants who are born preterm develops within the more noxious environment of the neonatal intensive care unit (NICU), which is often characterized by high sound and light levels and by inconsistent caregiving patterns [6–10]. Moreover, even when efforts are made to improve NICU caregiving using developmental care models, the prevalence of poor neurodevelopmental outcomes is high and the incidence appears to be increasing, perhaps due to the complex relationships among biologic and environmental risks, caregiving behaviors, and the timing, size, and site of brain insults.

Few interventions have been shown to reduce neurobehavioral disorganization and poor neurodevelopment in children who were born preterm. Improvement in neurodevelopmental outcomes depends on interventions that take advantage of the preterm infant’s capabilities [11]. Further, it is important that relationships among multiple risk factors, interventions, and outcomes are articulated so that interventions can be tailored specifically to infants with varying risk profiles [12].

Our intervention, a patterned feeding experience (PFE), is not currently used in most neonatal settings. The intervention is based on principles of neural plasticity and neuroprotection and is designed to take advantage of neuronal synaptic development and both experience-expectant and experience-dependent characteristics of the developing brain [13]. In addition, the intervention takes advantage of a regularly occurring caregiving and life-sustaining event in the life of an infant–feeding. The PFE intervention involves a continuity of tactile experiences associated with feeding to train and build neuronal networks supportive of the normal experience. There are virtually no accepted and no tested caregiver behaviors related to enteral feedings in preterm infants, although some recent research has produced evidence for a positive effect of greater caregiver sensitivity to the infant during feeding [14]. Theoretically, our intervention provided at a critical time in neurologic system development and associated with a recurring event such as feeding should enhance neural connections that may be important for later development, particularly language and other cognitive and neurobehavioral organization skills [15].

The overall objective of this study is to test the effect of a PFE from birth to NICU discharge for infants born at 32 weeks gestation and younger. The primary aim of the study is to test the effect of a PFE on neurobehavioral organization at 3 time points: the transition to oral feedings, NICU discharge, and 2 months corrected age (48 weeks PMA). Secondary aims are to test the effect of a PFE on 1) cognitive function at 6 months corrected age; 2) neurobehavioral development; and 3) clinical outcomes including time to first oral feeding, time to full oral feeding, and time to discharge. An exploratory aim is to describe the potential effects of perinatal and infant risk factors including immunologic and genetics influences on the outcome variables (neurobehavioral organization, cognitive function, neurobehavioral development, and clinical outcomes). We hypothesize that infants who receive the PFE will have improved neurobehavioral organization, cognitive function, neurobehavioral development, and clinical outcomes compared to infants who receive usual feeding care in the NICU.

## Methods

### Design

The study is a longitudinal randomized control trial with two groups of preterm infants, one receiving PFE from the first gavage feeding through full oral feedings to discharge and one receiving usual feeding experience. Ethical approval for all aspects of the study was obtained from the Institutional Review Board, Cincinnati Children’s Hospital Medical Center (2011–0871). A data and safety monitoring plan is in place and a data and an independent safety monitoring board meets biannually to review trial conduct including assessment of any adverse events. All study data are kept confidential and in stored in secure data bases. The protocol is guided by the Standard Protocol Items: Recommendations for Interventional Trials (SPIRIT) [16], 2013 statement, which can be found in Additional file 1, and the Consolidated Standards of Research Trials (CONSORT) [17].

### Setting

The study is being conducted in two NICUs that are staffed by the same physician group, thus providing similarity in medical care related to feeding practices. Nurses at the two institutions are organizationally separate and, thus, there are differences in nursing practices. Post-discharge data collection for all enrolled infants occurs in a high-risk infant follow-up clinic which provides comprehensive ongoing services to high-risk infants through an interdisciplinary team.

### Sample

One hundred and twenty preterm infants (60/group) will be enrolled. Infants are included if they are less ≤ 32 weeks PMA at birth and parents provide informed written consent. Infants are excluded if they have known gastrointestinal, craniofacial, cardiovascular, neuromuscular, and/or genetic defects. Infants of both genders and all ethnic and racial backgrounds are recruited. We expect approximately 5 % of the sample to be Hispanic and 50 % to be Black/African American. Approximately 10 % of the sample is expected to be multiple gestation (twin or triplet). Two to three infants are enrolled per month; this number is based on data from the clinical setting, our experiences in previous studies, the length of the intervention, and our data collection processes. Infants who are medically unable to transition to oral feeding by 40 weeks PMA are excluded from further study.

Infants are recruited by research nurses who are experienced in neonatal care and trained in study procedures. Initial contact is generally made within 24 to 48 hours after the infant’s birth. A study brochure is provided to parents and a demonstration of study procedures using a doll is given. Parents are encouraged to think about participation for as long as they need; study nurses usually contact parents within 1 to 2 days of first contact to obtain parents’ decision.

A randomization scheme was created by the study statistician for study group assignment using Statistical Analysis Software (SAS 9.3, SAS Institute Inc., Cary, NC, USA). The random group assignments are placed on a card and sealed, only to be used after an infant was enrolled. Once enrolled, infants are randomly assigned to either the intervention or the control group by drawing a card from the assignment set. Parents of twins are allowed to request a single randomization card for both twins or to allow their infants to be randomized independently. Only one twin from a twinset where both infants were randomized with the same card will be included in the analysis.

#### Power analysis

The proposed sample size for the study is based on associations between measures of feeding experience and components of neurobehavioral development and neurobehavioral organization discovered in our previous studies [18–21]. We anticipate 20 % attrition, primarily for medical conditions that arise or are diagnosed after enrollment. The sample size was determined by assuming each aim will be tested using 2-sided tests at *P* = 0.05. For the primary aim, we expect to detect an effect size of at least 0.7 standard deviation (SD) between groups on the final neurobehavioral score, with at least 90 % power using a 2-group *t*-test. However, a random effects model that takes into account the repeated measurements over time is proposed for the analyses of neurobehavior. Differences of the same effect size should improve the power to at least 95 % because of the additional number of observations. Although we have powered the study to test the primary aim, we also considered power for the additional aims. For secondary aim 1 involving a test of the effect of the intervention on cognitive function at 6 months, the same sample size analysis indicates a 2-unit difference (SD = 3) on cognitive development will be detected with 89 % power with only a 2-group *t*-test; the analysis of covariance (ANCOVA) should have more power. For secondary aim 2, the analyses and the sample size calculations are the same as with the primary aim. Secondary aim 3 will be tested in a manner similar to secondary aim 1 and so the sample size calculations should be similar. For the exploratory aim a sample of 96 infants in a test for zero correlation will provide at least 90 % power to detect *r* ≥ 0.26 and at least 80 % power to detect *r* ≥ 0.21.

#### Attrition management

We provide a US$25 incentive to the parents of all participating infants when the infant begins oral feeding and at hospital discharge. We also provide US$25 for each post-discharge visit at 2 and 6 months corrected age. To further enhance retention, we maintain contact with families via Email and postcards to keep up-to-date on addresses and telephone numbers and changing circumstances related to infant health. We send follow-up reminders immediately after discharge to promote higher subject retention and we offer flexible scheduling for the post-discharge visits.

### Intervention

The study intervention has two phases using a tactile experience designed to pattern the neurologic system’s theoretical expectation for feeding experience. Phase 1, a period of “attended gavage,” begins within 24 hours of the first gavage feeding, which is generally provided before 72 hours of life. Attended gavage includes tactile containment during the entire gavage infusion by gravity plus 5 minutes. For infants too ill to be removed from the incubator, tactile containment consists of gentle hand containment (placing one hand lightly over the shoulder area and one hand lightly over the lower extremities). If medically stable, the infant is swaddled and removed from the incubator and held. Infants are also offered nonnutritive sucking (NNS) as part of every attended gavage feeding based on prior research showing that NNS facilitates behavioral organization. Instructions related to the tactile containment, swaddling/holding during gavage, and use of NNS are provided for all nursing staff and parents of infants in the intervention group. Gavage feedings are most often provided on a schedule of every 3 hours. Infants on continuous feedings, which may be needed for some infants primarily because of gastric reflux, receive the attended protocol every 3 hours for 15 minutes. Phase 1 of the intervention has varying duration, lasting as long as 12 weeks for infants born at 23 weeks PMA. Attended gavage is a change in practice for most NICUs so we use a team of well-trained study nurses who provide the attended gavage feedings for a minimum of 50 % of the feedings daily. Nursing staff or parents may provide additional attended gavage feedings. We record the occurrence of each attended feeding including who provided the intervention (parent, nursing staff member, or study nurse) and include that “dose” as a covariate in our analyses.

Phase 2 begins when infants in the intervention group begin oral feedings. When an oral feeding is attempted, the infant is held outside the incubator in a swaddled, flexed position either upright or side-lying. We record the volume of feeding prescribed and the amount consumed at each feeding. Infants who are unable to complete the prescribed volume orally or unable to orally feed receive attended gavage as described in Phase 1.

In both Phase 1 and Phase 2, parents of infants in the intervention group are encouraged to participate by holding their infants for feedings. Feeding at the breast is encouraged for all infants whose mothers have planned to breast feed and the use of breast milk is encouraged for all feedings. Intervention fidelity is monitored on a quarterly basis to ensure that infants in the intervention group receive a minimum intervention dose of 50 %.

### Usual care

Infants in the usual care group do not receive the tactile intervention during gavage feedings, including those that occur when the infant is in the transition period from gavage to oral feeding. When infants receiving usual care are gavage fed, they are almost always in the incubator and they are not handled or touched during the feeding. While the infant may be in a “nested” position using positioning devices, nurses rarely swaddle infants, use their hands to contain infant movements, or hold infants during gavage feedings. Occasionally these infants are swaddled and held by their parents during gavage feedings. However, this occurs only rarely and without any consistency. We are recording all occurrences of any tactile exposure usual care, control group infants receive during gavage feedings.

### Measures

Neurobehavioral organization will be measured using the Neurobehavioral Assessment of the Preterm Infant (NAPI) [22]. Cognitive function will be measured using the Bayley Scales of Infant and Toddler Development, Third Edition, (BSID III) [23]. Neurobehavioral development will be measured using sucking organization, feeding performance, and heart rate variability. Clinical outcomes are measured using data obtained from the health record. Data for risk factors associated with neurodevelopmental outcomes are obtained from the health record or from biologic samples obtained specifically for the study.

#### Neurobehavioral organization

The NAPI is used to measure neurobehavioral organization. The NAPI measures seven domains of neurobehavior and assesses the effect of NICU interventions. It is a non-intrusive, objective assessment of the infant’s maturity of functioning with construct validity with neonatal morbidity. We will use mean scores for each domain in our analyses with particular attention to the alertness and orientation score. The NAPI can be administered with the infant in an incubator or open crib and can be used if the infant is receiving oxygen; some items are not used if the infant is on mechanical ventilation. The assessment is done at least 30 minutes before feeding and uses a standardized format maximizing the opportunity to test various functions in appropriate behavior states. Most of the exam is observational and does not require handling the infant. The NAPI is administered at baseline, transition to oral feeding and NICU discharge assessments at the infant’s bedside by an experienced NAPI examiner. The NAPI assessment at 2 months corrected age is completed by the same examiners at the follow-up clinic. The NAPI is predictive of early developmental delay and later motor development, neurobehavior, and cognition; NAPI scores on alertness and orientation skills at 36 weeks PMA predict BSID III outcomes.

#### Cognitive function

The BSID III is a widely used measure of development in infants and toddlers. The BSID III consists of five scales of development: Cognitive, Language, Motor, Social-Emotional, and Adaptive Behavior scales; we are most interested in Cognitive scale results. The Cognitive scale contains 91 items that provide information about attention and anticipatory behavior, exploration of environment, object retention, cause and effect, object permanence, relational play, imitation, grouping, classification, memory, and problem solving. Administration time for children ≤ aged 12 months is less than 50 minutes. Raw scores from the Cognitive scale are converted to a scaled score, which can then additionally be converted to a composite score equivalent. Percentile ranks, confidence intervals (90 % and 95 % levels), growth score equivalents, and developmental age scores in months and days are available. The internal consistency for the Cognitive scale is 0.91. The BSID III is administered at 6 months corrected age by a trained, blinded examiner.

#### Prenatal risk factors

Maternal risk factors associated with neurodevelopmental outcomes include premature rupture of the membranes, pregnancy-induced hypertension, antenatal steroid or selective serotonin re-uptake inhibitor treatment, parity, chorioamnionitis, or diabetes mellitus [24, 25]. These data are included in the infant’s record and will be recorded after enrollment. Fetal or infant risk factors are intrauterine growth restriction, low 5-minute Apgar score, metabolic or respiratory acidosis within the first 24 hours of life, sepsis as defined by positive cultures, and evidence of intraventricular hemorrhage or periventricular leukomalacia on early and late cranial ultrasounds, which are routinely obtained in our setting at 2 weeks of life and at 34 to 36 weeks PMA [26, 27]. Data on these risk factors are available in the infant’s medical record and will be recorded by the study nurses. We will collect data for computing severity of illness using the Neonatal Medical Index (NMI), a tool we have used in previous studies [28]. The NMI summarizes infants’ medical condition with classifications ranging from 1 for infants born weighing ≥ 1000 g and without major complications to 5 for infants born weighing < 1000 g and with very serious complications. The scale distinguishes between the neurobehavioral performance of infants with varying degrees of illness and predicts developmental progress at 12, 24, and 36 months of age.

#### Immunologic measures

We collect blood within 7 days after birth in conjunction with a medically indicated blood draw for measurement proinflammatory cytokines. Nurses in the study setting draw the blood and place it in transport bags that have been left at the bedside. Study nurses check for samples each morning and transport samples to our laboratory. The laboratory uses a Bio-Plex (Bio-Rad, Hercules, CA, USA) multiplex suspension array system capable of measuring up to 100 different analytes simultaneously in small (12-μL) amounts of fluid in 1 well of a 96-well plate. Thus, only a small sample (0.5 mL) of blood is needed. Plasma and lymphocytes are separated using standard Ficoll gradient methods, and samples are divided into aliquots, frozen, and stored at −70 °C for batch assays. The assay accurately measures cytokine values in the range of 1–2500 pg/mL is precise (intra-assay coefficient of variability (CV) < 10 %, interassay CV < 15 %) and shows less than 1 % cross-reactivity among cytokines or with other molecules. Based on current literature and our own experienced, we use a customized panel to assay for cytokines of interest, including IL-1ra, IL-6, IL-8, IL-10, granulocyte-colony stimulating factor (GCSF), granulocyte macrophage colony stimulating factor (GMCSF), IFN-γ, monocyte chemotactic protein-1 (MCP-1), and TNF-α. All work with biohazardous materials is done in accordance with established laboratory safety guidelines.

#### Genetic measures

We collect saliva when infants are at the transition from gavage to oral feeding for genetic analysis using OrageneDNA® (DNA Genotek Inc., Kanata, Ontario, Canada), a collection kit that is non-invasive, intuitive to use, and stabilizes DNA at elevated temperatures, which facilitates transport and storage. Samples are frozen and stored for later analysis. We have selected a small number of theoretically and empirically determined genetic markers to assess including *ApoE* genotype (*ApoEε3*, *ApoEε2*, and *ApoEε4*) and the candidate genes mesenchymal epithelial transition factor (*MET*), neuregulin 3 (*NRG3*), and solute carrier family 6 member 4 (*SLC6A4*) [29]. We will use Applied Biosystems’ StepOnePlus® RT-PCR instrument and TaqMan® SNP genotyping assays (Life Technologies, ThermoFisher Scientific, Grand Island, NY, USA).

#### Neurobehavioral development

Both CNS development and autonomic nervous system (ANS) function are measured as indicators of neurobehavioral development. Sucking organization (suck bursts/feeding, burst duration, interburst interval) is a reliable measure of CNS function [30]. In addition, oral feeding performance (proportion consumed, efficiency) is a reliable indicator of CNS integrity [31]. These measures are taken during twice weekly observation feedings during Phase 2 of the intervention or, for infants in the usual care group, once oral feedings have started. To measure sucking, a noninvasive, piezoelectric sensor is placed on the infant’s jaw where it detects movements associated with sucking. A computer data acquisition system based on the Biopac Systems MP-150® data acquisition system is used to collect and store data (BIOPAC Systems, Inc, Goleta, CA, USA). Software developed by our team allows extraction and processing of the data from the acquisition file using algorithms developed by us.

Feeding performance consists of two measures: proportion consumed and efficiency. These measures have been reliably used in numerous feeding studies [32–34]. Proportion consumed, which incorporates both oral-motor skill and level of endurance, is the percent of prescribed formula or breast milk consumed over the feeding time, not counting breaks for burping or rest. The amount consumed is recorded at the end of the feeding. Efficiency refers to the total volume taken over feeding time and is a reflection of oral-motor skill as well as fatigue. The amount taken is recorded by the study nurse at the end of the observation feeding. Feeding time is calculated by the data acquisition system and eliminates non-feeding times (that is burping or rest breaks).

Heart rate variability (HRV) is a measure of ANS maturation and an indication of infant well-being and development [35, 36]. HRV increases with increasing PMA and, as the infant is more able to control breathing, an increase in parasympathetic activity occurs, as seen in the high frequency (HF) power spectrum of HRV. Both parasympathetic and sympathetic activity is seen in the low frequency (LF) power spectrum. Higher respiratory rates and activity result in a higher LF:HF ratio, consistent with a stress model. HRV effects have been found to be associated with poor autonomic organization during feedings. We examine HRV twice a week during observation feedings with data collected by the acquisition system described above. The electrocardiogram waveform is sampled at a rate of 1000 samples/second; the instantaneous R-R time interval is used for computation of HRV after being digitally filtered to remove artifact. HRV includes the LF and HF proportions (in normalized units) and the LF:HF ratio.

#### Clinical outcomes

Time to full oral feedings, the duration (in days) between the initiation of oral feeding and the achievement of full oral feeding, and length of stay, the number of days between the start of oral feeding and discharge to home, are also measured. Full oral feedings are achieved when the infant is consuming all nutrients orally by breast or bottle without intravenous supplementation or gavage feeding for 24 hours.

#### Demographic data

Birth weight and gestation, race, ethnicity, and gender are recorded from the infant’s medical record by the study nurses at the time of infant enrollment. Maternal demographic data are also collected including age, race, ethnicity and parity.

### Procedures

Admissions to the NICU are monitored daily by the study nurses who contact parents in person about the study when their infant is 24 to 48 hours old; most mothers are still in hospital at this time. Informed consent discussions take place in the private rooms with one of the study nurses, all of whom are experienced NICU nurses skilled in talking to families about preterm infants. Research equipment is available during the consent discussion and a doll is used to show placement of sensors. Parents are encouraged to handle equipment and ask questions. After obtaining written informed consent, the study nurse gathers demographic data and sets up the data collection file. Each infant is assigned a code number. The files are kept locked in the research setting with daily transfer to the study research office for safe storage.

#### Randomization to groups

A random assignment scheme was developed by the study statistician using a random number generator. Infants are assigned to groups at enrollment in equal numbers using 120 cards marked with one or other group contained in sealed envelopes and kept in a locked cabinet in the study office. Assignment to group is not blinded to parents, NICU staff, or the study nurses. The examiner for the major study outcomes is blinded to group assignment.

#### Data collection procedures

The principal investigator and the study nurses provide in-service instruction for unit staff at both study settings. Data collection is completed by the study nurses; however, NICU staff are instructed in how to provide the intervention for infants in the intervention group and to maintain appropriate clinical records. Parents and other family members of infants in the intervention group also receive instruction in providing the intervention.

### Observation feedings

The study nurses schedule observation feedings twice a week during Phase 2 using procedures developed in our previous studies [22]. Observation feedings are scheduled to be compatible with parent and nursing staff schedules. These feedings generally are scheduled at times parents are not present since ideally parents prefer to feed their infant and we do not wish to interfere with this important process. Parents may be present during an observation feeding but they do not participate in these feedings; since observation feedings occur only twice a week and only during Phase 2, this is not difficult to achieve. During observation feedings, the computer data acquisition system continuously records heart rate and sucking activity. The study nurse oversees data acquisition to ensure that clear signals are obtained; the electronic file can also be “marked” using pre-assigned event keys for changes in position and movements, stops for burps, and rest periods made at the discretion of the feeder. Study nurses also record the volume consumed for computation of feeding performance measurements of efficiency and percent consumed. Observation feedings involve two people–a nurse feeding the baby (feeder) and a study nurse.

Routine vital signs and basic care (e.g., diaper change) are completed by the study nurse who then applies the research equipment. The infant remains on the unit’s monitoring equipment for oxygen saturation, respiration and heart rate. The infant is loosely swaddled in a blanket in a flexed position and removed from the incubator. Once the nurse is seated with the infant the data acquisition system is started, providing a continuous recording of HR and sucking activity.

Experienced neonatal nurses who are trained in the feeding procedures feed the infants. The feeder will feed the infant using the prescribed formula or breast milk in a volufeeder. Lighting and sound levels in the room are recorded as these may affect infants’ feeding performance. The feeding continues until the infant completes the prescribed amount, has no sucking activity for 2 minutes, or shows signs of distress (e.g., bradycardia), whichever occurs first. The infant is offered burps and rest periods at the discretion of the nurse. When the infant has completed the feeding or the feeding is halted, the time is recorded and the data acquisition system stopped. The infant is returned to the incubator and research equipment removed. The amount of fluid remaining in the bottle is recorded for computer computation of feeding skill efficiency and percent consumed. If the infant does not consume the prescribed volume during feeding, the study nurse gives the remaining amount by gavage using Phase 1 procedures appropriate for group assignment.

### Data management and analysis

A relational database has been created. Monthly assessment of data entry processes and a quarterly audit of random samples for completeness are conducted. During the electronic data acquisition process, all sampled data from each of the physiologic channels are stored in a single file. To prepare these data for analysis, the data are digitally filtered to remove frequencies not part of the physiologic signal spectrum. The filtered signal is then applied to our algorithms resulting in the relevant parameters.

Descriptive statistics will be computed for all demographic data to describe the sample and check for missing and outlying data. Descriptive statistics will also be computed for all component measures of study variables. Transformations of non-normally distributed variables will be used for analyses as indicated. Because measures are repeated on the same infant, all analyses will take into account the dependency between measures. Because some infants may be unable to complete the study due to unexpected illness, there will be an “all-infants” dataset (all eligible) and a “per-protocol” dataset (all completers). An intent-to-treat analysis will be performed on the all-infants dataset using all measures obtained. An analysis on the “per-protocol” dataset will be performed using the data from the infants who completed the entire study. All results will be reported as described by the CONSORT guidelines [17]. Analyses will be performed using SAS (SAS Institute Inc., Cary, NC, USA) at an overall alpha = 0.05 significance level.

The primary aim states that infants who receive the PFE will have improved neurobehavioral organization at transition to oral feedings, NICU discharge, and 2 months corrected age (48 weeks PMA). The difference between the PFE and usual care groups on the NAPI will be tested using a repeated-measures ANCOVA. A mixed-model will account for the correlation between the measurements at each time point and allow for differential patterns of missing values at the three time points. A significant difference across these time points will be followed by separate comparisons at each time point; each will be performed at the *P* = 0.05 level of significance.

The secondary aims require similar comparisons between study groups on secondary outcomes (cognitive functioning, neurobehavioral development, and clinical outcomes). To test secondary aim 1, differences between the 2 groups on the cognitive outcome measure (BSIDIII cognitive subtest scaled score) at 6 months corrected age will be tested using an ANCOVA with morbidity (NMI) and birth gestation used as covariates. To test secondary aim 2, 4 analyses will be performed, one for each of the feeding-related neurobehavioral outcomes (sucking organization, 2 measures of feeding performance, and HRV). The modeling will be done as above with a repeated-measures mixed-model approach with covariates (birth gestation, morbidity). To test secondary aim 3, separate ANCOVA analyses of the clinical outcomes (time to full oral feeding and length of stay) will be used to compare the 2 groups with the birth gestation age and morbidity covariates.

The intent of the exploratory aim is to identify risk factors of poor neurobehavioral development and cognitive dysfunction in preterm infants. We will test the assumed comparability of randomly assigned groups prior to undertaking further analyses. Secondary analyses will focus on the interactions between risk factors and the intervention differences by first screening the risk factors for effects at *P* = 0.2 and then developing a single exploratory model that includes all of the risk factors that significantly influence the outcome differences between the groups. Additional analyses will focus on the findings for neurobehavioral development including sucking organization and feeding performance, HRV, and clinical outcomes, first by examining the relationships between the risk factors and the components of neurobehavioral development and then by assessing the relationships among the components of neurobehavioral development and the primary outcome measure, the NAPI score (neurobehavioral outcome). The final goal is to identify a model of risk factors, modifiers (measures of neurobehavioral development), and mediators (intervention) that have an effect on the differences between groups.

## Discussion

This study is a randomized trial to test the effectiveness of a theoretically derived, neuroprotective intervention for preterm infants developed to be easily incorporated into the context of routine NICU care. The effect of the intervention on preterm infant neurobehavioral organization, cognitive development and neurodevelopment as well as on clinical outcomes will be measured. We will also assess the potential effect of various risk factors on the neurodevelopmental outcomes and as potential moderators of intervention effectiveness. The results of the study have the potential to change neonatal feeding practices, which currently do not systematically support infant neurologic development. Moreover, the study results are potentially applicable to other groups of hospitalized infants including those requiring surgery shortly after birth.

The intervention has several strengths including its strong theoretical link to experience- dependent neural development. The intervention costs little and is easily implemented in a busy NICU. Moreover, the intervention can be provided by family members, nurses and other care providers with minimal training, making its implementation and long-term sustainability more likely.

Study results will be reported to participating study settings, the National Institutes of Health, professional organizations via investigator presentations, and to the scientific and lay public via journal publications.

## Trial status

Recruiting participants.

## Additional file

Additional file 1:
**SPIRIT checklist.**

## Abbreviations

ANCOVA: analysis of covariance
ANS: autonomic nervous system
*ApoE*: apolipoprotein E
BSID III: Bayley Scales of Infant and Toddler Development, Third Edition
CNS: central nervous system
CONSORT: Consolidated Standards of Reporting Trials
CV: coefficient of variability
GCSF: granulocyte-colony stimulating factor
GMCSF: granulocyte macrophage colony stimulating factor
HF: high frequency
HRV: heart rate variability
IFN-γ: interferon gamma
IL: interleukin
LF: low frequency
MCP-1: monocyte chemotactic protein-1
*MET*: mesenchymal epithelial transition factor
NAPI: Neurobehavioral Assessment of the Preterm Infant
NICU: neonatal intensive care unit
NMI: Neonatal Medical Index
NNS: nonnutritive sucking
*NRG3*: neuregulin 3
PFE: patterned feeding experience
PMA: post-menstrual age
RT-PCR: reverse transcription polymerase chain reaction
SAS: Statistical Analysis Software
SD: standard deviation
*SLC6A4*: solute carrier family 6 member 4
SNP: single nucleotide polymorphism
SPIRIT: Standard Protocol Items: Recommendations for Interventional Trials
TNF-α: tumor necrosis factor alpha
